# The Cost of Provisions in Institutions.—X

**Published:** 1904-06-25

**Authors:** 


### HOSPITAL ADMINISTRATION.

### CONSTRUCTION AND ECONOMICS.

### THE COST OF PROVISIONS IN INSTITUTIONS.—X.

By the Author of "Hospital Expenditure—The Commissariat."

(*Continued from page* 212.)

A COMPARISON.

The foregoing articles on the cost of provisions in some of the leading London hospitals have rendered possible a detailed comparison of expenditure on the Hospital Commissariat for the year 1902.

In order to compare to any useful purpose the gross cost of provisions per bed, the number and constitution of the household must be clearly set forth in relation to the number of beds. We propose then, in the first place, to show in order of numbers the inmates other than patients boarded in those hospitals whose accounts we have scrutinised.

We have placed the hospitals in order according to the proportion of inmates other than patients boarded in the institution. It is obvious that provisions will cost more per bed in proportion to the numbers in the household, even although such increased cost may be overbalanced by differences in price and administration. The proportion of non-patients is shown in two ways. First, by dividing the occupied beds among them—the range is from 1·1 to 1·8 apiece. Secondly, by giving the percentage which non-patients form of the total number of inmates boarded—the range is from 45 to 35 per cent. The cost of provisions is next divided between patients and non-patients. And since the number of those other than patients is not appreciably affected by fluctuations in the numbers of occupied beds, the cost of the household, exclusive of patients, has been calculated first on the number of occupied beds, and next on the number of available beds. A careful study of these figures will reveal the fact that the cost per occupied bed decreases in fairly regular proportion according to the rate of the numbers other than patients. Passing over the Great Northern Central, where the domestic management is exceptionally successful, the range of cost, after the board of the patients has been deducted, is from £16¼ at St. George's per available bed, to £9½ at the Royal Free. The rate per occupied bed at St. George's appears excessive, owing to the fact that the average of occupied beds in the hospital was only 251 in 1902 as compared with an ordinary average of about 300. But allowing this fact its full weight, it will be seen that the same tendency to decrease is observable in the calculations based on the number of occupied beds. There are several striking points of similarity in expenditure between hospitals differing widely in size and domestic arrangements.

Table I.

| Hospital | Daily average of occupied beds | Daily average of inmates other than patients | Average of occupied beds divided among non-patients boarded in hospital | | | | Non-patients' percentage of total number of inmates | Cost of provisions | | | Remarks |
|---|---|---|---|---|---|---|---|---|---|---|---|
| | | | | | | | | Patients only | Exclusive of patients | | |
| | | | Doctors | Nurses | House | Total | | Per occupied bed | Per occupied bed | Per available bed | |
| | | | | | | | | £ s. | £ | £ | |
| St. George's .. .. | 251 | 213 | 19·3 | 1 7 | 5 | 1·1 | 45 | 14 15 | $22\frac{3}{4}$ | $16\frac{1}{4}$ | Ten wards closed for repairs in 1902 |
| University College .. | 168 | 126 | 14 | 2 | 7 | 1·3 | 43 | 13 0* | 17* | 15* | * Approximate. |
| St. Mary's .. .. | 248 | 166 | 13 | 2·5 | 5 | 1·4 | 40 | — | — | — | |
| Charing Cross .. .. | 132 | 87 | 13·2 | 2·5 | 5 | 1·5 | 40 | 16 0* | $12\frac{1}{2}$* | $11\frac{1}{4}$* | * Approximate. |
| Guy's .. .. .. | 485 | 317 | 28 | 2 | 7·3 | 1·5 | 39 | 16 0 | 13 | $9\frac{3}{4}$ | |
| Great Northern Central | 137 | 83 | 15 | 3·3 | 4·1 | 1·6 | 38 | 11 10 | $8\frac{1}{2}$ | $7\frac{1}{4}$ | |
| Royal Free .. .. | 144 | 81 | 26 | 2·9 | 9·6 | 1·8 | 35 | 17 0 | $11\frac{1}{4}$ | 10 | |
| St. Thomas's .. .. | 465 | 244 | 27 | 2·4 | 7·2 | 1·9 | 33 | 12 3 | $11\frac{1}{5}$ | $9\frac{1}{2}$ | |

In the next table we have placed together, so far as this can be ascertained, the cost of boarding the various elements of the hospital household. There is a remarkable difference in the cost of boarding the patient extending from the minimum of 7½d. a day at the Great Northern Central, and 8¾d. at St. Thomas's, to the maximum of 11¼d. at the Royal Free, and amounting to as much as £5 a year on each occupied bed. Such a difference amounts to £1,000 a year on every 200 beds. It does not appear that £17 a year is by any means an excessive sum for the patients' board. The average is £16 at Charing Cross and at Guy's, and the domestic management at the Royal Free Hospital is notably excellent. It is because we see these differences occur under skilled management that we begin to understand how money may run away when no one in authority attempts to check the bills or find out exactly what it costs to victual the household.

After a careful comparison of diet sheets, some of which we have quoted in previous articles, we have reached the conclusion that hospital managers are entirely in accord as to the proper diet for ordinary patients. It is impossible to take one diet-sheet and say that it would work out at greater expense than another. The hospitals enumerated all provide tea, sugar, and butter for their patients. The proportion of patients on low or fever diet, who cost very little, seem, so far as can be ascertained, to be much the same in all; and children, who are less expensive to feed than adults, form in all these hospitals only a small proportion of the patients. The variation in cost, though it may depend partly on prices, is in all probability a question of "extras." These

TABLE II.—*Cost of Boarding per Head.*

| Hospital | Patients | | Doctors | Nurses | House | All inmates |
|---|---|---|---|---|---|---|
| | Per year | Per day | Per Year | Per year | Per year | Per year |
| | £ s. | d. | £ | £ s. | £ s. | £ s. |
| St. George's .. .. .. .. .. .. .. | 14 15 | 9¾ | 60 | 17 14 | — | 20 6 |
| University College .. .. .. .. .. .. | *13 0 | *8½ | 55 | 18 5 | *16 0 | 17 2 |
| Charing Cross .. .. .. .. .. .. .. | 16 0 | 10¼ | — | *17 0 | — | 17 0 |
| Guy's .. .. .. .. .. .. .. | 16 0 | 10½ | 75 | 17 4 | 17 4 | 17 15 |
| without Bright's Ward .. .. .. .. .. | 14 15 | 9¾ | — | — | — | — |
| Great Northern Central .. .. .. .. .. | 11 10 | 7¼ | — | — | †14 0 | 12 10 |
| St. Thomas's .. .. .. .. .. .. | 12 13 | 8⅓ | 55 | 23 0 | *13 0 | 15 12 |
| Royal Free .. .. .. .. .. .. .. | 17 0 | 11¼ | 52 | 19 0 | — | 18 2 |
| St. Mary's .. .. .. .. .. .. .. | — | — | — | — | — | — |

* Approximate. † Including doctors, nurses, servants.

variations of diet, ordered by the surgeon or physician in charge of the case, may be said in familiar phrase to drive a coach and four through the diet-sheets and upset all domestic theories of economy at will.

To certain persons, and perhaps generally to the public at large, it may seem niggardly and unfeeling to limit the extent to which "extras" are supplied to sick people. But when it is remembered that the patient is in residence for cure, and that with well-understood exceptions, he gets well as quickly without "extras" as with them, the supposed hardship of keeping him on rather rigid and monotonous diet for 21 days melts away.

The cost of the medical officers' board varies between £1 and £1 10s. a week. At Guy's Hospital, where the higher figure is reached, the amount is paid out for board, and therefore includes service, fuel, lighting, rent, etc., in addition to the cost of the food, which is alone included in the other estimates.

The cost of boarding the nursing staff varies from about 6s. 6d. to 8s. 10d. a week.

We append a third table, which may be of service to hospital managers. It gives a calculation of the cost per occupied bed and per head, including all inmates, for the various items of provisions enumerated in the published reports. Difference in prices here rises into prominence. The actual amount of meat consumed per head appears to vary but little, and the quantity of milk required in the house and among the patients is much the same in the instances where it has been ascertained. Yet, to give but one example, the cost per head for milk shows a difference of 16s. as between St. Thomas's and St. George's Hospitals, and there is an even greater difference in the cost of meat. We have found the numbers boarded at St. Thomas's to be 709, and the saving to the hospital on these two items alone as compared with St. George's Hospital, is over £1,300.

TABLE III.

| Hospital | Meat | | Fish and Poultry | | Bacon, etc. | | Eggs | | Milk | | Bread and Flour | | Grocery | | Vegetables | | Malt Liquors | | Ice and Mineral Waters | Wine and Spirits | Remarks |
|---|---|---|---|---|---|---|---|---|---|---|---|---|---|---|---|---|---|---|---|---|---|
| | Per Head | Per Bed | Per Head | Per Bed | Per Head | Per Bed | Per Head | Per Bed | Per Head | Per Bed | Per Head | Per Bed | Per Head | Per Bed | Per Head | Per Bed | Per Head | Per Bed | Per Bed | Per Bed | |
| | £ s. | £ s. | £ s. | £ s. | £ s. | £ s. | £ s. | £ s. | £ s. | £ s. | £ s. | £ s. | £ s. | £ s. | £ s. | £ s. | £ s. | £ s. d. | £ s. | £ s. | |
| St. George's .. .. | 6 2 | — | 2 1 | — | 2 8 | — | 1 1 | — | 3 14 | — | 1 4 | — | 1 15 | — | 1 0 | — | 0 14 | — | 0 4 | 0 16 | |
| Univ. Coll. (patients', officers, and household) | 5 8 | 10 8 | 2 1 | *3 15 | 1 9 | — | 0 16 | — | 2 16 | *4 8 | 0 18 | *1 13 | 1 11 | — | 1 0 | *2 0 | 0 6 | — | 0 8 | 0 17 | * Including nurses also |
| Univ. Coll. (nurses only) | 7 6 | — | 2 5 | — | — | — | — | — | 1 14 | — | 1 1 | — | — | — | 1 9 | — | — | — | — | — | |
| St. Mary's .. .. | 5 3 | 8 12 | 2 10 | 4 6 | 2 5 | 3 15 | 1 13 | 2 15 | 2 17 | 4 16 | 1 2 | 1 17 | 1 12 | 2 13 | 0 15 | 1 6 | 0 10 | 0 18 0 | 0 4 | 1 0 | |
| Charing Cross .. .. | 6 0 | 9 17 | 1 7 | 2 5 | 2 3 | 3 11 | 0 11 | 0 18 | 2 16 | 4 14 | 1 5 | 2 1 | 1 6 | 2 4 | 0 14 | 1 4 | 0 8 | 0 14 0 | 0 7 | 0 17 | |
| Guy's (patients).. .. | — | 4 12 | — | 2 0 | — | 0 19 | — | 0 17 | — | 3 3 | — | 1 4 | — | 2 13 | — | 0 9 | — | 0 0 11 | 0 12 | 0 16 | |
| Guy's (nurses and household .. .. .. | 4 15 | — | 0 18 | — | 4 5 | — | 0 8 | — | 1 12 | — | 0 17 | — | 2 7 | — | 1 8 | — | 0 4 | — | — | — | |
| Great Northern Central | 4 10 | 7 5 | 0 16 | 1 6 | 1 4 | 2 0 | 0 11 | 0 18 | 2 12 | 4 3 | 0 18 | 1 10 | 1 6 | 2 2 | 0 8 | 0 13 | 0 3 | 0 2 0 | 0 8 | 1 2 | |
| St. Thomas's .. .. | 5 4 | 7 18 | 1 9 | 2 5 | 1 15 | 2 13 | 0 9 | 0 14 | 2 16 | 4 8 | 0 19 | 1 10 | 2 2 | 3 5 | 0 13 | 1 0 | 0 7 | 0 11 0 | 0 4 | 0 6 | |
| Royal Free .. .. | 4 14 | 7 7 | 2 5 | 4 12 | 2 15 | 3 18 | 0 19 | 1 9 | 3 3 | 4 18 | 1 0 | 1 12 | 1 16 | 2 16 | 0 19 | 1 9 | 0 3 | 0 4 0 | 0 10 | 0 9 | |

